# Laplacian Estrada and Normalized Laplacian Estrada Indices of Evolving Graphs

**DOI:** 10.1371/journal.pone.0123426

**Published:** 2015-03-30

**Authors:** Yilun Shang

**Affiliations:** 1 Department of Mathematics, Tongji University, Shanghai, China; Nankai University, CHINA

## Abstract

Large-scale time-evolving networks have been generated by many natural and technological applications, posing challenges for computation and modeling. Thus, it is of theoretical and practical significance to probe mathematical tools tailored for evolving networks. In this paper, on top of the dynamic Estrada index, we study the dynamic Laplacian Estrada index and the dynamic normalized Laplacian Estrada index of evolving graphs. Using linear algebra techniques, we established general upper and lower bounds for these graph-spectrum-based invariants through a couple of intuitive graph-theoretic measures, including the number of vertices or edges. Synthetic random evolving small-world networks are employed to show the relevance of the proposed dynamic Estrada indices. It is found that neither the static snapshot graphs nor the aggregated graph can approximate the evolving graph itself, indicating the fundamental difference between the static and dynamic Estrada indices.

## Introduction

With the development of modern digital technologies, time-dependent complex networks arise naturally in a variety of areas from peer-to-peer telecommunication to online human social behavior to neuroscience. The edges in these networks, which represent the interactions between elements of the systems, change over time, posing new challenges for modeling and computation [1, 2]. Basically, the time ordering of the networks (or graphs) induces an asymmetry in terms of information communication, even though each static snapshot network is symmetric, i.e., undirected [3]. For example, if *u* communicates with *v*, and then later *v* communicates with *w*, the information from *u* can reach *w* but not vice versa.

The Estrada index as a graph-spectrum-based invariant, on the other hand, was put forward by Estrada [4], initially for static graphs. Since its invention in 2000, the Estrada index has found a range of applications in chemistry and physics, including the degree of folding of long-chain polymeric molecules (especially proteins) [4, 5], extended atomic branching [6], and vibrations in complex networks [7–10], etc. The Estrada index of a graph *G* with *n* vertices is defined as [11]

$$\begin{array}{c} EE\left(G\right)=\sum_{i=1}^{n}e^{\lambda_{i}}, \end{array}$$ (1)

where *λ*_1_, *λ*_2_, ⋯, *λ*_*n*_ are the eigenvalues of the adjacency matrix of *G*. Numerous mathematical results for the Estrada index have been obtained, especially the upper and lower bounds. For these results, we refer the reader to an updated review [12] and the references therein. From the combinatorial construction, it is easy to see that *EE*(*G*) counts the weighted sum of closed walks of all lengths in *G*. The Estrada index—viewed as a redundancy measure of alternative paths—is shown to be instrumental in gauging robustness of networks [9, 13–17].

However, all the above mentioned works on the Estrada index are only confined to static graphs, which is a drawback from the perspective of network science [2]. Very recently, the Estrada index of time-dependent networks is introduced in [18] based on a natural definition of a walk on an evolving graph, namely, a time-ordered sequence of graphs over a fixed vertex set. Given an evolving graph, this dynamic Estrada index respects the time-dependency and generalizes the (static) Estrada index, conveniently summarizing those networks. Some basic properties and lower and upper bounds for the dynamic Estrada index are also developed in [18].

In the present paper, we go deeper in this direction and consider the dynamic Laplacian Estrada index and the dynamic normalized Laplacian Estrada index. In addition to the spectrum of adjacency matrix, the spectral theory of (normalized) Laplacian matrix is another well developed part in algebraic graph theory [19, 20]. We show that it is possible to define dynamic (normalized) Laplacian Estrada index in full analogy with dynamic Estrada index [18]. In fact, the static Laplacian Estrada and normalized Laplacian Estrada indices have already been proposed in [21] and [22], respectively. As such, our work can be viewed as an extension from static case to dynamic case. The gap between them, nevertheless, is non-trivial as described at the outset.

After giving the two dynamic indices and some basic properties, we establish refined upper and lower bounds for them, respectively. All these bounds are presented in terms of the several simplest graph-theoretic parameters, such as the numbers of vertices (or nodes) and edges, and the maximum and minimum degrees, offering both conceptual and computational advantages. Moreover, the similarity and difference between dynamic Estrada index and dynamic (normalized) Laplacian Estrada index are explored. In some cases, the dynamic (normalized) Laplacian Estrada index behaves better than its counterpart due to the nice properties of Laplacian spectrum [20].

Next, we use synthetic examples (random evolving small-world networks) to validate the relevance of our proposed various dynamic Estrada indices. Simulation results highlight the fundamental difference between the static and dynamic Estrada indices—in general, neither the static snapshot graphs nor the aggregated/summarized graph approximates the evolving graph itself.

We mention here that there is an increasing interest in studying evolving graphs in recent few years. The most conceptually relevant works are [3, 23–25], where static Katz-like centralities and network communicability are accommodated to address the time-evolving scenarios. A continuous-time dynamical systems view of node centrality in evolving networks is provided in [26]. However, these works are mostly concerned about algorithmic aspects, such as computational cost, efficiency and storage. We also note that the evolving networks have found a place in the analysis of coevolutionary games and more broadly, the emergence of cooperation in complex adaptive systems [27–29].

## Results

### Concepts of dynamic Estrada indices

We first review the dynamic Estrada index [18] and then introduce the related concepts of dynamic (normalized) Laplacian Estrada indices with some general properties.

Let *G* be a simple graph with *n* vertices. Denote by *A* = *A*(*G*) the adjacency matrix of *G*, and *λ*_1_(*A*), *λ*_2_(*A*), ⋯, *λ*_*n*_(*A*) the eigenvalues of *A*. Since *A* is a real symmetric matrix, we assume that the eigenvalues are labeled in a non-increasing manner as *λ*_1_(*A*) ≥ *λ*_2_(*A*) ≥ ⋯ ≥ *λ*_*n*_(*A*). Let tr(⋅) represent the trace of a matrix. For *k* = 0, 1, ⋯, define $M_{k}(A)=\sum_{i=1}^{n}\lambda_{i}^{k}(A)$ the *k*th spectral moment of the adjacency matrix. It follows from (1) that the Estrada index of *G* can be written as

$$\begin{array}{c} EE\left(G\right)=\sum_{i=1}^{n}e^{\lambda_{i}\left(A\right)}=\sum_{k=0}^{\infty }\frac{M_{k}\left(A\right)}{k!}=\sum_{k=0}^{\infty }\frac{\mathrm{tr}(A^{k})}{k!}=\mathrm{tr}\left(e^{A}\right), \end{array}$$ (2)

where the power-series expansion of matrix exponential *e* ^*A*^ is employed:

$$\begin{array}{c} e^{A}=I+A+\frac{A^{2}}{2!}+\cdots +\frac{A^{k}}{k!}+\cdots =\sum_{k=0}^{\infty }\frac{A^{k}}{k!} \end{array}$$ (3)

with *I* being the *n*-dimensional identity matrix. An extension to weighted graphs can be found in [30].

Suppose we have an evolving graph, namely, a time-ordered sequence of simple graphs *G*_1_, *G*_2_, ⋯, *G*_*N*_ over a fixed set *V* of *n* vertices, at the time points 1, 2, ⋯, *N*. Let *A*_*t*_ = *A*(*G*_*t*_) be the adjacency matrix for the snapshot graph *G*_*t*_ for *t* = 1, 2, ⋯, *N*. Let *m*_*t*_ denote the number of edges of *G*_*t*_ and *λ*_1_(*A*_*t*_) ≥ *λ*_2_(*A*_*t*_) ≥ ⋯ ≥ *λ*_*n*_(*A*_*t*_) the eigenvalues of *A*_*t*_.

**Definition 1**. [18] The Estrada index of an evolving graph *G*_1_, *G*_2_, ⋯, *G*_*N*_ is defined as

$$\begin{array}{c} EE\left(G_{1},G_{2},\cdots ,G_{N}\right)=\mathrm{tr}\left(e^{A_{1}}e^{A_{2}}\cdots e^{A_{N}}\right). \end{array}$$ (4)

The following concept of dynamic walk in an evolving graph is introduced in [3].

**Definition 2**. A dynamic walk of length *k* from vertex *v*_1_ ∈ *V* to vertex *v*_*k*+1_ ∈ *V* consists of a sequence of edges {*v*_1_, *v*_2_}, {*v*_2_, *v*_3_}, ⋯, {*v*_*k*_, *v*_*k*+1_} and a non-decreasing sequence of time points 1 ≤ *t*_1_ ≤ *t*_2_ ≤ ⋯ ≤ *t*_*k*_ ≤ *N* such that the (*v*_*i*_, *v*_*i*+1_) element of *A*_*t*_*i*__, (*A*_*t*_*i*__)_*v*_*i*_, *v*_*i*+1__ ≠ 0 for 1 ≤ *i* ≤ *k*.

In the light of (3), the product of matrix exponentials $e^{A_{t_{1}}}e^{A_{t_{2}}}\;\cdots \;e^{A_{t_{k}}}$ is equal to the summation of all products of the form

$$\begin{array}{c} \frac{1}{\eta_{1}!\eta_{2}!\cdots \eta_{k}!}A_{t_{1}}^{\eta_{1}}A_{t_{2}}^{\eta_{2}}\cdots A_{t_{k}}^{\eta_{k}}=\frac{1}{\eta_{1}!\eta_{2}!\cdots \eta_{k}!}A_{t_{s_{1}}}^{\delta_{1}}A_{t_{s_{2}}}^{\delta_{2}}\cdots A_{t_{s_{r}}}^{\delta_{r}}, \end{array}$$

where *t*_*s*_1__ < *t*_*s*_2__ < ⋯ < *t*_*s*_*r*__ are all the distinct values in the time sequence *t*_1_ ≤ *t*_2_ ≤ ⋯ ≤ *t*_*k*_, and the multiplicity of *t*_*s*_*i*__ is *δ*_*i*_, namely, *δ*_*i*_ = ∑_*t*_*j*_ = *t*_*s*_*i*___ *η*_*j*_, 1 ≤ *i* ≤ *r*. Note that the matrix product *A*_*t*_1__ *A*_*t*_2__ ⋯ *A*_*t*_*k*__ has (*v*_*p*_, *v*_*q*_) element that counts the number of dynamic walks of length *k* from *v*_*p*_ to *v*_*q*_ on which the *i*th step of the walk takes place at time *t*_*i*_, 1 ≤ *i* ≤ *k*. Thus, by setting $\ell ≔\sum_{i=1}^{r}\delta_{i}=\sum_{j=1}^{k}\eta_{j}$, we observe that the dynamic Estrada index (4) is a weighted sum of the numbers of closed dynamic walks of all lengths, where the number of walks of length ℓ (with *δ*_*i*_ edges followed at time *t*_*s*_*i*__, 1 ≤ *i* ≤ *r*) is penalized by a factor $\frac{1}{\eta_{1}!\eta_{2}!\;\cdots \;\eta_{k}!}$, naturally extending the (static) Estrada index (2).

#### Dynamic Laplacian Estrada index

Given a simple *n*-vertex graph *G*, its degree matrix *D*(*G*) is defined as a diagonal matrix with degrees of the corresponding vertices of *G* on the main diagonal and zero elsewhere. The Laplacian matrix of *G* is *L* = *L*(*G*) ≔ *D*(*G*) − *A*(*G*). We assume that *λ*_1_(*L*) ≥ *λ*_2_(*L*) ≥ ⋯ ≥ *λ*_*n*_(*L*) = 0 are the Laplacian eigenvalues of *G* [20].

The Laplacian analogue of the Estrada index is defined in [21] as

$$\begin{array}{c} LEE\left(G\right)=\sum_{i=1}^{n}e^{\lambda_{i}\left(L\right)}. \end{array}$$ (5)

An essentially equivalent definition can be found in [31]. We refer the reader to [32–34] for recent results of *LEE*(*G*) and its variants. For *k* = 0, 1, ⋯, define $M_{k}(L)=\sum_{i=1}^{n}\lambda_{i}^{k}(L)$ the *k*th spectral moment of the Laplacian matrix. Then, the expression (5), in parallel with (2), implies that

$$\begin{array}{c} LEE\left(G\right)=\sum_{i=1}^{n}e^{\lambda_{i}\left(L\right)}=\sum_{k=0}^{\infty }\frac{M_{k}\left(L\right)}{k!}=\sum_{k=0}^{\infty }\frac{\mathrm{tr}(L^{k})}{k!}=\mathrm{tr}\left(e^{L}\right), \end{array}$$

which elicits the following dynamic Laplacian Estrada index:

**Definition 3**. The Laplacian Estrada index of an evolving graph *G*_1_, *G*_2_, ⋯, *G*_*N*_ is defined as

$$\begin{array}{c} LEE\left(G_{1},G_{2},\cdots ,G_{N}\right)=\mathrm{tr}\left(e^{L_{1}}e^{L_{2}}\;\cdots \;e^{L_{N}}\right), \end{array}$$ (6)

where *L*_*t*_ = *L*(*G*_*t*_), *t* = 1, 2, ⋯, *N*.

For two simple graphs *G* and *H* over the same vertex set *V*, we define their weighted union as an edge-weighted graph *G* ⊔ *H* with adjacency matrix (*A*(*G* ⊔ *H*))_*u*, *v*_ = 2 if {*u*, *v*} appears in both *G* and *H*, and (*A*(*G* ⊔ *H*))_*u*, *v*_ = 1 if {*u*, *v*} appears in just one of *G* and *H*. For an integer *N* > 0, let $G^{\left(N\right)}≔\underset{N\;\text{multiples}}{\underset{︸}{G⊔G\;\cdots \;⊔G}}$ for short. Some elementary mathematical properties of the dynamic Laplacian Estrada index can be drawn straightforwardly:

1^∘^ Denote by *S*_*N*_ be the symmetric group of order *N*. It follows from the cyclic property of trace, that, for *N* ≤ 3,
  $$\begin{array}{c} LEE\left(G_{1},G_{2},\cdots ,G_{N}\right)=LEE\left(G_{\sigma (1)},G_{\sigma (2)},\cdots ,G_{\sigma (N)}\right),\;\sigma \in S_{N}, \end{array}$$
  and that, for general *N*,
  $$\begin{array}{c} LEE\left(G_{1},G_{2},\cdots ,G_{N}\right)=LEE\left(G_{N},G_{1},\cdots ,G_{N-1}\right)=\cdots =LEE\left(G_{2},G_{3},\cdots ,G_{1}\right). \end{array}$$
  This invariance under cyclic permutation also holds for the dynamic Estrada index [18].
2^∘^ As a direct consequence of (6), if $G_{N}=\bar{K_{n}}$, the (edgeless) complement of complete graph *K*_*n*_, then
  $$\begin{array}{c} LEE\left(G_{1},G_{2},\cdots ,G_{N}\right)=LEE\left(G_{1},G_{2},\cdots ,G_{N-1}\right). \end{array}$$
  The same also holds for the dynamic Estrada index [18].
3^∘^ Suppose that *G*_1_ = *G*_2_ = ⋯ = *G*_*N*_. Then
  $$\begin{array}{c} LEE\left(G_{1},G_{2},\cdots ,G_{N}\right)=LEE\left(G_{1}^{\left(N\right)}\right). \end{array}$$
  Similarly, we have $EE(G_{1},G_{2},\cdots ,G_{N})=EE(G_{1}^{\left(N\right)})$.
4^∘^ If *G*_1_ = *G*_2_ = ⋯ = *G*_*N*_ is an *r*-regular bipartite graph. Then
  $$\begin{array}{c} LEE\left(G_{1},G_{2},\cdots ,G_{N}\right)=e^{rN}\cdot EE\left(G_{1}^{\left(N\right)}\right). \end{array}$$
  The property 4^∘^ can be seen as follows.
  $$\begin{array}{ccc} LEE(G_{1},G_{2},\cdots ,G_{N}) & = & \mathrm{tr}\left(e^{N(rI-A_{1})}\right)=e^{rN}\mathrm{tr}\left(e^{-NA_{1}}\right)\\ & = & e^{rN}\mathrm{tr}\left(e^{NA_{1}}\right)=e^{rN}\cdot EE\left(G_{1}^{\left(N\right)}\right), \end{array}$$
  where in the second last equality we used the fact that the eigenvalues of *A*_1_ are symmetric around zero [20]. Note that the static case *N* = 1 corresponds to [21, Prop. 6(d)] or [31, Lem. 4].

#### Dynamic normalized Laplacian Estrada index

The normalized Laplacian matrix ℒ = ℒ(*G*) is defined as [19]

$$\begin{array}{c} {\left(ℒ\right)}_{i,j}=\left\{\begin{array}{cc} 1, & i=j,\;\deg_{G}\left(v_{i}\right)\neq 0;\\ -\frac{1}{\sqrt{\deg_{G}\left(v_{i}\right)\deg_{G}\left(v_{j}\right)}}, & i\neq j,v_{i}\;\mathrm{is}\;\mathrm{adjacent}\;\mathrm{to}\;v_{j};\\ 0, & \mathrm{otherwise}, \end{array}\right. \end{array}$$

where deg_*G*_(*v*_*i*_) is the degree of vertex *v*_*i*_ in *G*. If there is no isolated vertex in *G*, we have ℒ(*G*) = *D* ^−1/2^(*G*)*L*(*G*)*D* ^−1/2^(*G*). Assume that *λ*_1_(ℒ) ≥ *λ*_2_(ℒ) ≥ ⋯ ≥ *λ*_*n*_(ℒ) = 0 are the normalized Laplacian eigenvalues of *G*.

The normalized Laplacian Estrada index is put forward in [35] as

$$\begin{array}{c} ℒEE\left(G\right)=\sum_{i=1}^{n}e^{\lambda_{i}\left(ℒ\right)}. \end{array}$$ (7)

See also [22] for an essentially equivalent definition. ℒ*EE*(*G*) has been addressed for a class of tree-like fractals [36]. Following the same reasoning in (2), we obtain ℒ*EE*(*G*) = tr(*e* ^ℒ^). In analogy to (4) and (6), we have the following

**Definition 4**. The normalized Laplacian Estrada index of an evolving graph *G*_1_, *G*_2_, ⋯, *G*_*N*_ is defined as

$$\begin{array}{c} ℒEE\left(G_{1},G_{2},\cdots ,G_{N}\right)=\mathrm{tr}\left(e^{{ℒ}_{1}}e^{{ℒ}_{2}}\;\cdots \;e^{{ℒ}_{N}}\right), \end{array}$$ (8)

where ℒ_*t*_ = ℒ(*G*_*t*_), *t* = 1, 2, ⋯, *N*.

The following basic properties of the dynamic normalized Laplacian Estrada index can be easily deduced.

5^∘^ For *N* ≤ 3,
  $$\begin{array}{c} ℒEE\left(G_{1},G_{2},\cdots ,G_{N}\right)=ℒEE\left(G_{\sigma (1)},G_{\sigma (2)},\cdots ,G_{\sigma (N)}\right),\;\sigma \in S_{N}, \end{array}$$
  and, for general *N*,
  $$\begin{array}{c} ℒEE\left(G_{1},G_{2},\cdots ,G_{N}\right)=ℒEE\left(G_{N},G_{1},\cdots ,G_{N-1}\right)=\cdots =ℒEE\left(G_{2},G_{3},\cdots ,G_{1}\right). \end{array}$$
6^∘^ If $G_{N}=\bar{K_{n}}$,
  $$\begin{array}{c} ℒEE\left(G_{1},G_{2},\cdots ,G_{N}\right)=ℒEE\left(G_{1},G_{2},\cdots ,G_{N-1}\right). \end{array}$$
7^∘^ Suppose that *G*_1_ = *G*_2_ = ⋯ = *G*_*N*_. Then
  $$\begin{array}{c} ℒEE\left(G_{1},G_{2},\cdots ,G_{N}\right)=\sum_{i=1}^{n}e^{N\lambda_{i}\left({ℒ}_{1}\right)}, \end{array}$$
  whereas $ℒEE(G_{1}^{\left(N\right)})=ℒEE(G_{1})$.
8^∘^ If *G*_1_ = *G*_2_ = ⋯ = *G*_*N*_ is an *r*-regular bipartite graph (*r* ≥ 1). Then
  $$\begin{array}{c} ℒEE\left(G_{1},G_{2},\cdots ,G_{N}\right)<e^{N}\cdot EE^{1/r}\left(G_{1},G_{2},\cdots ,G_{N}\right)=e^{N}EE^{1/r}\left(G_{1}^{\left(N\right)}\right). \end{array}$$

To see 8^∘^, we have

$$\begin{array}{ccc} ℒEE(G_{1},G_{2},\cdots ,G_{N}) & = & \mathrm{tr}\left(e^{N{ℒ}_{1}}\right)=\sum_{i=1}^{n}e^{N(r-\lambda_{i}\left(A_{1}\right))/r}=e^{N}\sum_{i=1}^{n}{\left(e^{-N\lambda_{i}\left(A_{1}\right)}\right)}^{1/r}\\ & \leq & e^{N}{\left(\sum_{i=1}^{n}e^{-N\lambda_{i}\left(A_{1}\right)}\right)}^{1/r}\\ & = & e^{N}{\mathrm{tr}}^{1/r}\left(e^{NA_{1}}\right)=e^{N}\cdot EE^{1/r}\left(G_{1}^{\left(N\right)}\right), \end{array}$$

where the equality is attained if and only if *λ*_2_(ℒ_1_) = ⋯ = *λ*_*n*_(ℒ_1_) = 0. This condition is equivalent to $G_{1}=\bar{K_{n}}$ or $G_{1}=K_{2}\cup \bar{K_{n-2}}$, which contradicts the assumption. Theorem 3.4 in [35] can be reproduced by setting *N* = 1.

### Bounds for dynamic Laplacian Estrada index

**Proposition 1**. *Let G*_1_, *G*_2_, ⋯, *G*_*N*_ *be an evolving graph over a set V of size n. Then*

(i) $LEE(G_{1},G_{2},\cdots ,G_{N})\leq {\left(\prod_{t=1}^{N}LEE\left(G_{t}^{\left(N\right)}\right)\right)}^{1/N}\leq \frac{1}{N}\sum_{t=1}^{N}LEE(G_{t}^{\left(N\right)})$.
*The equalities are attained if and only if G*_1_ = *G*_2_ = ⋯ = *G*_*N*_.
(ii) max{*LEE*(*G*_1_), *LEE*(*G*_2_)} ≤ *LEE*(*G*_1_, *G*_2_) ≤ min{*e* ^*λ*_1_(*L*_1_)^ *LEE*(*G*_2_), *e* ^*λ*_1_(*L*_2_)^ *LEE*(*G*_1_)}.
*The equalities are attained if and only if* $G_{1}=\bar{K_{n}}$ or $G_{2}=\bar{K_{n}}$.

**Proof**. (i) Since the matrices ${\left\{e^{L_{t}}\right\}}_{t=1}^{N}$ are positive definite, it follows from the extended Bellman inequality ([37, p. 481] or [38]) that

$$\begin{array}{ccc} LEE(G_{1},G_{2},\cdots ,G_{N}) & = & \mathrm{tr}(e^{L_{1}}e^{L_{2}}\;\cdots \;e^{L_{N}})\\ & \leq & {\left(\prod_{t=1}^{N}\;\mathrm{tr}\left(e^{NL_{t}}\right)\right)}^{1/N}={\left(\prod_{t=1}^{N}LEE\left(G_{t}^{\left(N\right)}\right)\right)}^{1/N}. \end{array}$$

The last inequality follows from the arithmetic-geometric means inequality. Both equalities are attained if and only if *G*_1_ = *G*_2_ = ⋯ = *G*_*N*_.

(ii) Note that

$$\begin{array}{c} LEE\left(G_{1},G_{2}\right)=\mathrm{tr}\left(e^{L_{1}}e^{L_{2}}\right)=\mathrm{tr}\left(e^{\frac{1}{2}L_{1}}e^{L_{2}}e^{\frac{1}{2}L_{1}}\right). \end{array}$$

Therefore, *LEE*(*G*_1_, *G*_2_) ≥ e^λ_*n*_(*L*_1_)^tr(*e* ^(L_2_)^ = *LEE*(*G*_2_) since *λ*_*n*_(*L*_1_) = 0, and *LEE*(*G*_1_, *G*_2_) ≤ e^λ_1_(*L*_1_)^tr(*e* ^(L_2_^) = *e* ^*λ*_1_(*L*_1_)^ *LEE*(*G*_2_). Since *λ*_*i*_(*L*_1_) = 0 for all *i* is equivalent to $G_{1}=\bar{K_{n}}$, the above two equalities hold if and only if $G_{1}=\bar{K_{n}}$. The desired result then follows from the property 1^∘^.

**Remark**. By counting the number of closed walks, it is shown in [18, Prop. 1] that

$$\begin{array}{c} EE\left(G_{1},G_{2},\cdots ,G_{N}\right)\geq \sum_{i=1}^{N}EE\left(G_{i}\right). \end{array}$$ (9)

However, this does not hold for *LEE* even in the case of *N* = 2. To see this, we take $G_{1}=\bar{K_{n}}$. Then,

$$\begin{array}{c} LEE\left(G_{1},G_{2}\right)=LEE\left(G_{2}\right)<LEE\left(G_{1}\right)+LEE\left(G_{2}\right). \end{array}$$

Recall that *m*_*t*_ is the number of edges in *G*_*t*_, *t* = 1, 2, ⋯, *N*.

**Proposition 2**. *The Laplacian Estrada index of an evolving graph G*_1_, *G*_2_, ⋯, *G_N_ over a set of n vertices with N* = 2 *is bounded by*

$$\begin{array}{c} \frac{1}{2}+\sqrt{\frac{1}{4}+n\left(n-1\right)e^{\frac{4(m_{1}+m_{2})}{n}}}\leq LEE\left(G_{1},G_{2}\right)\leq n-1+\frac{1}{2}\left(e^{4m_{1}}+e^{4m_{2}}\right). \end{array}$$

*The equality on the left-hand side is attained if and only if* $G_{1}=G_{2}=\bar{K_{n}}$; *and the equality on the right-hand side is attained if and only if* $G_{1}=G_{2}=\bar{K_{n}}$ *or* $G_{1}=G_{2}=K_{2}\cup \bar{K_{n-2}}$.

**Proof**. *Lower bound*. Based on the well-known Golden-Thompson inequality (see e.g. [38]) we obtain

$$\begin{array}{c} LEE\left(G_{1},G_{2}\right)=\mathrm{tr}\left(e^{L_{1}}e^{L_{2}}\right)\geq \mathrm{tr}\left(e^{L_{1}+L_{2}}\right). \end{array}$$

Therefore,

$$\begin{array}{ccc} LEE^{2}\left(G_{1},G_{2}\right) & \geq & {\left(\sum_{i=1}^{n}e^{\lambda_{i}\left(L_{1}+L_{2}\right)}\right)}^{2}\\ & = & \sum_{i=1}^{n}e^{2\lambda_{i}\left(L_{1}+L_{2}\right)}+2\sum_{1\leq i<j\leq n}e^{\lambda_{i}\left(L_{1}+L_{2}\right)}e^{\lambda_{j}\left(L_{1}+L_{2}\right)}. \end{array}$$ (10)

Using Proposition 1 (i), we obtain

$$\begin{array}{ccc} LEE(G_{1},G_{2}) & \leq & \frac{1}{2}\left(LEE\left(G_{1}^{\left(2\right)}\right)+LEE\left(G_{2}^{\left(2\right)}\right)\right)=\frac{1}{2}\sum_{i=1}^{n}\left(e^{2\lambda_{i}\left(L_{1}\right)}+e^{2\lambda_{i}\left(L_{2}\right)}\right)\\ & \leq & \sum_{i=1}^{n}e^{2\lambda_{i}\left(L\left(G_{1}⊔G_{2}\right)\right)}=\sum_{k=0}^{\infty }\sum_{i=1}^{n}\frac{{\left(2\lambda_{i}\left(L\left(G_{1}⊔G_{2}\right)\right)\right)}^{k}}{k!}\\ & = & n+4\left(m_{1}+m_{2}\right)+\sum_{k=2}^{\infty }\sum_{i=1}^{n}\frac{{\left(2\lambda_{i}\left(L\left(G_{1}⊔G_{2}\right)\right)\right)}^{k}}{k!}, \end{array}$$

where the second inequality comes from the interlacing theorem in which the equality holds if and only if $G_{1}=G_{2}=\bar{K_{n}}$. Note that *L*_1_ + *L*_2_ = *L*(*G*_1_ ⊔ *G*_2_). Then,

$$\begin{array}{ccc} \sum_{i=1}^{n}e^{2\lambda_{i}\left(L_{1}+L_{2}\right)} & = & \sum_{i=1}^{n}\sum_{k=0}^{\infty }\frac{{\left(2\lambda_{i}\left(L\left(G_{1}⊔G_{2}\right)\right)\right)}^{k}}{k!}\\ & = & n+4\left(m_{1}+m_{2}\right)+\sum_{k=2}^{\infty }\sum_{i=1}^{n}\frac{{\left(2\lambda_{i}\left(L\left(G_{1}⊔G_{2}\right)\right)\right)}^{k}}{k!}\\ & \geq & n+4\left(m_{1}+m_{2}\right)+LEE\left(G_{1},G_{2}\right)-n-4\left(m_{1}+m_{2}\right)\\ & = & LEE(G_{1},G_{2}). \end{array}$$ (11)

On the other hand, the arithmetic-geometric means inequality yields

$$\begin{array}{ccc} 2\sum_{1\leq i<j\leq n}e^{\lambda_{i}\left(L_{1}+L_{2}\right)}e^{\lambda_{j}\left(L_{1}+L_{2}\right)} & \geq & n\left(n-1\right){\left(\prod_{1\leq i<j\leq n}e^{\lambda_{i}\left(L\left(G_{1}⊔G_{2}\right)\right)}e^{\lambda_{j}\left(L\left(G_{1}⊔G_{2}\right)\right)}\right)}^{\frac{2}{n(n-1)}}\\ & = & n\left(n-1\right){\left({\left(\prod_{i=1}^{n}e^{\lambda_{i}\left(L\left(G_{1}⊔G_{2}\right)\right)}\right)}^{n-1}\right)}^{\frac{2}{n(n-1)}}\\ & = & n\left(n-1\right)e^{\frac{4(m_{1}+m_{2})}{n}}. \end{array}$$ (12)

Combining (10) with (11) and (12), we have

$$\begin{array}{c} LEE^{2}\left(G_{1},G_{2}\right)\geq LEE\left(G_{1},G_{2}\right)+n\left(n-1\right)e^{\frac{4(m_{1}+m_{2})}{n}}. \end{array}$$

Since ${\left(LEE\left(G_{1},G_{2}\right)-\frac{1}{2}\right)}^{2}\geq \frac{1}{4}+n(n-1)e^{\frac{4(m_{1}+m_{2})}{n}}\geq 0$, we arrive at

$$\begin{array}{c} LEE\left(G_{1},G_{2}\right)\geq \frac{1}{2}+\sqrt{\frac{1}{4}+n\left(n-1\right)e^{\frac{4(m_{1}+m_{2})}{n}}}. \end{array}$$

The equality is attained if and only if $G_{1}=G_{2}=\bar{K_{n}}$.

*Upper bound*. Since (*e* ^*L*_1_^ − *e* ^*L*_2_^)^2^ is a positive semi-definite matrix, we obtain

$$\begin{array}{ccc} 2LEE(G_{1},G_{2}) & = & 2\mathrm{tr}(e^{L_{1}}e^{L_{2}})\\ & \leq & \mathrm{tr}\left(e^{2L_{1}}\right)+\mathrm{tr}\left(e^{2L_{2}}\right)\\ & = & \sum_{i=1}^{n}\sum_{k=0}^{\infty }\frac{{\left(2\lambda_{i}\left(L_{1}\right)\right)}^{k}}{k!}+\sum_{i=1}^{n}\sum_{k=0}^{\infty }\frac{{\left(2\lambda_{i}\left(L_{2}\right)\right)}^{k}}{k!}\\ & = & n+2\sum_{i=1}^{n}\lambda_{i}\left(L_{1}\right)+2\sum_{i=1}^{n}\lambda_{i}^{2}\left(L_{1}\right)+\sum_{i=1}^{n}\sum_{k=3}^{\infty }\frac{{\left(2\lambda_{i}\left(L_{1}\right)\right)}^{k}}{k!}\\ & & +n+2\sum_{i=1}^{n}\lambda_{i}\left(L_{2}\right)+2\sum_{i=1}^{n}\lambda_{i}^{2}\left(L_{2}\right)+\sum_{i=1}^{n}\sum_{k=3}^{\infty }\frac{{\left(2\lambda_{i}\left(L_{2}\right)\right)}^{k}}{k!}\\ & = & 2n+4m_{1}+4m_{2}+2\left(Zg\left(G_{1}\right)+2m_{1}\right)+2\left(Zg\left(G_{2}\right)+2m_{2}\right)\\ & & +\sum_{k=3}^{\infty }\frac{1}{k!}\sum_{i=1}^{n}{\left(2\lambda_{i}\left(L_{1}\right)\right)}^{k}+\sum_{k=3}^{\infty }\frac{1}{k!}\sum_{i=1}^{n}{\left(2\lambda_{i}\left(L_{2}\right)\right)}^{k}, \end{array}$$

where $Zg(G)≔\sum_{i=1}^{n}\deg_{G}^{2}(v_{i})$ is called the first Zagreb index of graph *G* [39].

Note that $\sum_{i=1}^{n}{\left(2\lambda_{i}(L_{1})\right)}^{k}\leq {\left(\sum_{i=1}^{n}2\lambda_{i}(L_{1})\right)}^{k}$ with equality if and only if at most one of *λ*_1_(*L*_1_), *λ*_2_(*L*_1_), ⋯, *λ*_*n*_(*L*_1_) is non-zero, or equivalently, $G_{1}=\bar{K_{n}}$ or $G_{1}=K_{2}\cup \bar{K_{n-2}}$. Hence,

$$\begin{array}{ccc} 2LEE(G_{1},G_{2}) & \leq & 2n+8m_{1}+8m_{2}+2Zg\left(G_{1}\right)+2Zg\left(G_{2}\right)\\ & & +\sum_{k=3}^{\infty }\frac{1}{k!}{\left(\sum_{i=1}^{n}2\lambda_{i}\left(L_{1}\right)\right)}^{k}+\sum_{k=3}^{\infty }\frac{1}{k!}{\left(\sum_{i=1}^{n}2\lambda_{i}\left(L_{2}\right)\right)}^{k}\\ & = & 2n+8m_{1}+8m_{2}+2Zg\left(G_{1}\right)+2Zg\left(G_{2}\right)\\ & & +\sum_{k=3}^{\infty }\frac{1}{k!}{\left(4m_{1}\right)}^{k}+\sum_{k=3}^{\infty }\frac{1}{k!}{\left(4m_{2}\right)}^{k}\\ & = & 2n+4m_{1}+4m_{2}+e^{4m_{1}}+e^{4m_{2}}-8m_{1}^{2}-8m_{2}^{2}-2\\ & & +2(Zg\left(G_{1}\right)+Zg\left(G_{2}\right)). \end{array}$$ (13)

For *t* = 1, 2, denote by *n*_*t*_ the number of non-isolated vertices in *G*_*t*_. We have

$$\begin{array}{c} Zg\left(G_{t}\right)\leq \left(n_{t}-1\right)\sum_{i=1}^{n}\deg_{G_{t}}\left(v_{i}\right)\leq \left(2m_{t}-1\right)2m_{t}, \end{array}$$

with equality if and only if *G*_*t*_ = *K*_*n*_ or $G_{t}=K_{2}\cup \bar{K_{n-2}}$. Consequently,

$$\begin{array}{c} 2LEE\left(G_{1},G_{2}\right)\leq 2n+4^{4m_{1}}+e^{4m_{2}}-2, \end{array}$$

which yields the desired upper bound, in which equality is attained if and only if *G*_1_ = *G*_2_ = *K*_*n*_ or $G_{1}=G_{2}=K_{2}\cup \bar{K_{n-2}}$.

The previously communicated bounds for *EE*(*G*_1_, *G*_2_) in [18, Prop. 4] can not be attained. Here, we get tight bounds for *LEE*(*G*_1_, *G*_2_) thanks to the nice properties of Laplacian eigenvalues. We mention that a version of the thermodynamic inequality might also be used here [40, Lem. 1]. Let *δ*(*G*) and Δ(*G*) be the minimum and maximum degrees of graph *G*, respectively. We in the following establish new tight bounds with the help of the minimum and maximum degrees.

**Proposition 3**. *The Laplacian Estrada index of an evolving graph G*_1_, *G*_2_, ⋯, *G_N_ over a set V of n vertices with N* = 2 *is bounded by*

$$\begin{array}{ccc} & & \frac{1}{2}+\sqrt{\frac{1}{4}+n\left(n-1\right)e^{\frac{4(m_{1}+m_{2})}{n}}+\frac{8{\left(m_{1}+m_{2}\right)}^{2}}{n}+2n\delta_{12}\Delta_{12}-4\left(m_{1}+m_{2}\right)\left(\delta_{12}+\Delta_{12}\right)}\\ & & \;\leq LEE(G_{1},G_{2})\\ & & \;\leq n-1+\frac{1}{2}\left(e^{4m_{1}}+e^{4m_{2}}\right)+2m_{1}\left(1+\delta \left(G_{1}\right)+\Delta \left(G_{1}\right)\right)+2m_{2}\left(1+\delta \left(G_{1}\right)+\Delta \left(G_{1}\right)\right)\\ & & \;-4m_{1}^{2}-4m_{2}^{2}-n\delta \left(G_{1}\right)\Delta \left(G_{1}\right)-n\delta \left(G_{2}\right)\Delta \left(G_{2}\right), \end{array}$$

*where δ*_12_ ≔ *δ*(*G*_1_ ⊔ *G*_2_) *and* Δ_12_ ≔ Δ(*G*_1_ ⊔ *G*_2_). *The equalities are attained if and only if* $G_{1}=G_{2}=\bar{K_{n}}$.

**Proof**. *Lower bound*. As in the proof of Proposition 2, we have (10) and (12). In the following, we aim to obtain a new estimate involving *δ*_12_ and Δ_12_ for the first term on the right-hand side of (10).

We have

$$\begin{array}{ccc} \sum_{i=1}^{n}e^{2\lambda_{i}\left(L_{1}+L_{2}\right)} & = & \sum_{i=1}^{n}\sum_{k=0}^{\infty }\frac{{\left(2\lambda_{i}\left(L\left(G_{1}⊔G_{2}\right)\right)\right)}^{k}}{k!}\\ & = & n+4\left(m_{1}+m_{2}\right)+2\sum_{i=1}^{n}\lambda_{i}^{2}\left(L\left(G_{1}⊔G_{2}\right)\right)+\sum_{k=3}^{\infty }\sum_{i=1}^{n}\frac{{\left(2\lambda_{i}\left(L\left(G_{1}⊔G_{2}\right)\right)\right)}^{k}}{k!}\\ & = & n+8\left(m_{1}+m_{2}\right)+2Zg\left(G_{1}⊔G_{2}\right)+\sum_{k=3}^{\infty }\sum_{i=1}^{n}\frac{{\left(2\lambda_{i}\left(L\left(G_{1}⊔G_{2}\right)\right)\right)}^{k}}{k!}\\ & \geq & n+8\left(m_{1}+m_{2}\right)+\frac{8{\left(m_{1}+m_{2}\right)}^{2}}{n}+\sum_{k=3}^{\infty }\sum_{i=1}^{n}\frac{{\left(2\lambda_{i}\left(L\left(G_{1}⊔G_{2}\right)\right)\right)}^{k}}{k!} \end{array}$$ (14)

since

$$\begin{array}{c} Zg\left(G_{1}⊔G_{2}\right)=\sum_{v\in V}\deg_{G_{1}⊔G_{2}}^{2}\left(v\right)\geq \frac{1}{n}{\left(\sum_{v\in V}\deg_{G_{1}⊔G_{2}}\left(v\right)\right)}^{2}=\frac{4{\left(m_{1}+m_{2}\right)}^{2}}{n} \end{array}$$

by the Cauchy-Schwarz inequality. The equality holds if and only if *G*_1_ ⊔ *G*_2_ is regular.

By using Proposition 1 (i), as in the proof of Proposition 2, we obtain

$$\begin{array}{ccc} LEE(G_{1},G_{2}) & \leq & \sum_{k=0}^{\infty }\sum_{i=1}^{n}\frac{{\left(2\lambda_{i}\left(L\left(G_{1}⊔G_{2}\right)\right)\right)}^{k}}{k!}\\ & = & n+8\left(m_{1}+m_{2}\right)+2Zg\left(G_{1}⊔G_{2}\right)+\sum_{k=3}^{\infty }\sum_{i=1}^{n}\frac{{\left(2\lambda_{i}\left(L\left(G_{1}⊔G_{2}\right)\right)\right)}^{k}}{k!}\\ & \leq & n+8\left(m_{1}+m_{2}\right)+4\left(m_{1}+m_{2}\right)\left(\delta_{12}+\Delta_{12}\right)-2n\delta_{12}\Delta_{12}\\ & & +\sum_{k=3}^{\infty }\sum_{i=1}^{n}\frac{{\left(2\lambda_{i}\left(L\left(G_{1}⊔G_{2}\right)\right)\right)}^{k}}{k!}, \end{array}$$ (15)

where the last inequality follows from the fact

$$\begin{array}{c} Zg\left(G_{1}⊔G_{2}\right)\leq 2\left(m_{1}+m_{2}\right)\left(\delta_{12}+\Delta_{12}\right)-n\delta_{12}\Delta_{12} \end{array}$$

with equality attained if and only if *G*_1_ ⊔ *G*_2_ is a regular graph. Indeed, this can be seen by expanding the expression ∑_*v* ∈ *V*_(deg_*G*_1_ ⊔ *G*_2__(*v*) − *δ*_12_)(deg_*G*_1_ ⊔ *G*_2__(*v*) − Δ_12_), which is clearly non-positive.

Combining (10) with (12), (14) and (15), we obtain

$$\begin{array}{ccc} LEE^{2}\left(G_{1},G_{2}\right) & \geq & n+8\left(m_{1}+m_{2}\right)+\frac{8{\left(m_{1}+m_{2}\right)}^{2}}{n}+(LEE\left(G_{1},G_{2}\right)-n-8\left(m_{1}+m_{2}\right)\\ & & -\;4\left(m_{1}+m_{2}\right)\left(\delta_{12}+\Delta_{12}\right)+2n\delta_{12}\Delta_{12})+n\left(n-1\right)e^{\frac{4(m_{1}+m_{2})}{n}}\\ & = & \frac{8{\left(m_{1}+m_{2}\right)}^{2}}{n}+LEE\left(G_{1},G_{2}\right)-4\left(m_{1}+m_{2}\right)\left(\delta_{12}+\Delta_{12}\right)\\ & & +\;2n\delta_{12}\Delta_{12}+n\left(n-1\right)e^{\frac{4(m_{1}+m_{2})}{n}}. \end{array}$$

Therefore,

$$\begin{array}{ccc} {\left(LEE\left(G_{1},G_{2}\right)-\frac{1}{2}\right)}^{2} & \geq & \frac{8{\left(m_{1}+m_{2}\right)}^{2}}{n}-4\left(m_{1}+m_{2}\right)\left(\delta_{12}+\Delta_{12}\right)\\ & & +2n\delta_{12}\Delta_{12}+\frac{1}{4}+n\left(n-1\right)e^{\frac{4(m_{1}+m_{2})}{n}}\\ & \geq & 0, \end{array}$$ (16)

where the last inequality follows from the following three basic estimates:

$$\begin{array}{c} e^{\frac{4(m_{1}+m_{2})}{n}}\geq 1+\frac{4(m_{1}+m_{2})}{n},\;\Delta_{12}\leq n-1,\;\mathrm{and}\;2n\delta_{12}\Delta_{12}\geq 4\left(m_{1}+m_{2}\right)\delta_{12}. \end{array}$$

The desired lower bound then readily follows from (16).

*Upper bound*. As commented above, for *t* = 1, 2, we have

$$\begin{array}{c} Zg\left(G_{t}\right)\leq 2m_{t}\left(\delta \left(G_{t}\right)+\Delta \left(G_{t}\right)\right)-n\delta \left(G_{t}\right)\Delta \left(G_{t}\right), \end{array}$$

with equality attained if and only if *G*_*t*_ is a regular graph. Owing to (13), we obtain

$$\begin{array}{ccc} 2LEE(G_{1},G_{2}) & \leq & 2n+4m_{1}+4m_{2}+e^{4m_{1}}+e^{4m_{2}}-8m_{1}^{2}-8m_{2}^{2}-2\\ & & +2(Zg\left(G_{1}\right)+Zg\left(G_{2}\right))\\ & \leq & 2n+4m_{1}+4m_{2}+e^{4m_{1}}+e^{4m_{2}}-8m_{1}^{2}-8m_{2}^{2}-2\\ & & +4m_{1}\left(\delta \left(G_{1}\right)+\Delta \left(G_{1}\right)\right)-2n\delta \left(G_{1}\right)\Delta \left(G_{1}\right)\\ & & +4m_{2}\left(\delta \left(G_{2}\right)+\Delta \left(G_{2}\right)\right)-2n\delta \left(G_{2}\right)\Delta \left(G_{2}\right), \end{array}$$

which concludes the proof.

**Remark**. The bounds established in Proposition 2 and Proposition 3 are incomparable in general. In fact, for the lower bound, we note that

$$\begin{array}{c} \frac{8{\left(m_{1}+m_{2}\right)}^{2}}{n}\leq 4\left(m_{1}+m_{2}\right)\Delta_{12},\;\mathrm{but}\;2n\delta_{12}\Delta_{12}\geq 4\left(m_{1}+m_{2}\right)\delta_{12}; \end{array}$$

for the upper bound, we note that

$$\begin{array}{c} 2m_{t}\leq {\left(2m_{t}\right)}^{2},\;\mathrm{but}\;2m_{t}\left(\delta \left(G_{t}\right)+\Delta \left(G_{t}\right)\right)\geq n\delta \left(G_{t}\right)\Delta \left(G_{t}\right),\;t=1,2. \end{array}$$

We mention here that in the case of *N* = 1, some researchers bound the Laplacian Estrada index by using some more complicated graph-theoretic parameters, including graph Laplacian energy [31], namely, ∑_*i*_∣*λ*_*i*_(*L*)∣, and the first Zagreb index [41]. For more results on the graph energy, see e.g. [42–45]. The first Zagreb index was generalized to the zeroth-order general Randic index by Bollobás and Erdos [46], which was also useful in chemistry [47, 48]. In contrast, we only employ some of the most plain quantities to estimate the dynamic Laplacian Estrada index since (i) they are relatively easily accessible for real-life complex networks of interest to us, and (ii) our motivation comes from the potential application in gauging robustness for large-scale networks [9, 13, 16], where computational complexity matters.

### Bounds for dynamic normalized Laplacian Estrada index

The following proposition can be proved similarly as Proposition 1. Hence, we only state the result and omit its proof.

**Proposition 4**. *Let G*_1_, *G*_2_, ⋯, *G_N_ be an evolving graph over a set V of size n. Then*

(i) $ℒEE(G_{1},G_{2},\cdots ,G_{N})\leq {\left(\prod_{t=1}^{N}\text{tr}\left(e^{N{ℒ}_{t}}\right)\right)}^{1/N}\leq \frac{1}{N}\sum_{t=1}^{N}\text{tr}\left(e^{N{ℒ}_{t}}\right)$.
*The equalities are attained if and only if G*_1_ = *G*_2_ = ⋯ = *G*_*N*_.
(ii) max{ℒ*EE*(*G*_1_), ℒ*EE*(*G*_2_)} ≤ ℒ*EE*(*G*_1_, *G*_2_) ≤ min{*e* ^*λ*_1_(ℒ_1_)^ℒ*EE*(*G*_2_), *e* ^*λ*_1_(ℒ_2_)^ℒ*EE*(*G*_1_)}.
*The equalities are attained if and only if* $G_{1}=\bar{K_{n}}$ or $G_{2}=\bar{K_{n}}$.

**Remark**. The inequality (9) does not hold for ℒ*EE* either (even in the case of *N* = 2). To see this, we take $G_{1}=\bar{K_{n}}$. Then,

$$\begin{array}{c} ℒEE\left(G_{1},G_{2}\right)=ℒEE\left(G_{2}\right)<ℒEE\left(G_{1}\right)+ℒEE\left(G_{2}\right). \end{array}$$

**Proposition 5**. *The normalized Laplacian Estrada index of an evolving graph G*_1_, *G*_2_, ⋯, *G_N_ over a set of n* (*n* ≥ 2) *vertices with each snapshot graph being connected and N* = *2 is bounded by*

$$\begin{array}{c} \frac{2e^{2}}{e^{4}+1}\left(1+ne^{\frac{2n}{n-1}}\right)<ℒEE\left(G_{1},G_{2}\right)<e^{2}\left(n-1+e^{2\sqrt{n}}\right). \end{array}$$

**Proof**. *Lower bound*. From the well-known Neumann inequality, we obtain

$$\begin{array}{c} ℒEE\left(G_{1},G_{2}\right)=\mathrm{tr}\left(e^{{ℒ}_{1}}e^{{ℒ}_{2}}\right)\geq \sum_{i=1}^{n}e^{\lambda_{i}\left({ℒ}_{1}\right)}e^{\lambda_{n-i+1}\left({ℒ}_{2}\right)}. \end{array}$$

An elementary result of the normalized Laplacian eigenvalues [19] indicates that 1 ≤ *e* ^*λ*_*i*_(ℒ_1_)^ ≤ *e* ^2^ and 1 ≤ *e* ^*λ*_*i*_(ℒ_2_)^ ≤ *e* ^2^ for all 1 ≤ *i* ≤ *n*. Hence, applying an inverse of the Hölder inequality (see [37, p. 18] or [49]) gives

$$\begin{array}{c} ℒEE\left(G_{1},G_{2}\right)\geq \frac{2e^{2}}{e^{4}+1}\cdot {\left(\sum_{i=1}^{n}e^{2\lambda_{i}\left({ℒ}_{1}\right)}\right)}^{\frac{1}{2}}\cdot {\left(\sum_{i=1}^{n}e^{2\lambda_{i}\left({ℒ}_{2}\right)}\right)}^{\frac{1}{2}}. \end{array}$$ (17)

By the arithmetic-geometric means inequality, we obtain

$$\begin{array}{ccc} \sum_{i=1}^{n}e^{2\lambda_{i}\left({ℒ}_{1}\right)} & = & 1+\sum_{i=1}^{n-1}e^{2\lambda_{i}\left({ℒ}_{1}\right)}\\ & \geq & 1+e^{2\lambda_{1}\left({ℒ}_{1}\right)}+\left(n-2\right){\left(\prod_{i=2}^{n-1}e^{2\lambda_{i}\left({ℒ}_{1}\right)}\right)}^{\frac{1}{n-2}}\\ & = & 1+e^{2\lambda_{1}\left({ℒ}_{1}\right)}+\left(n-2\right)e^{\frac{2(n-\lambda_{1}\left({ℒ}_{1}\right))}{n-2}}, \end{array}$$ (18)

where in the last equality we used the equation $\sum_{i=1}^{n}\lambda_{i}({ℒ}_{1})=n$ since *G*_1_ is connected [19].

Define a function $f(x)≔1+2e^{2x}+(n-2)e^{\frac{2(n-x)}{n-2}}$. It is easy to check that $f^{\prime }(x)=4e^{2x}-2e^{\frac{2(n-x)}{n-2}}\geq 0$ if $x\geq \frac{2n-(n-2)\ln2}{2(n-1)}$. Since $\lambda_{1}({ℒ}_{1})\geq \frac{n}{n-1}\geq \frac{2n-(n-2)\ln2}{2(n-1)}$ for all *n* ≥ 2 [19], it follows from (18) that

$$\begin{array}{c} \sum_{i=1}^{n}e^{2\lambda_{i}\left({ℒ}_{1}\right)}\geq f\left(\lambda_{1}\left({ℒ}_{1}\right)\right)\geq f\left(\frac{n}{n-1}\right)=1+ne^{\frac{2n}{n-1}}. \end{array}$$ (19)

Likewise, we have

$$\begin{array}{c} \sum_{i=1}^{n}e^{2\lambda_{i}\left({ℒ}_{2}\right)}\geq 1+ne^{\frac{2n}{n-1}}. \end{array}$$ (20)

Combining these with (17) gives the desired lower bound

$$\begin{array}{c} ℒEE\left(G_{1},G_{2}\right)\geq \frac{2e^{2}}{e^{4}+1}\left(1+ne^{\frac{2n}{n-1}}\right). \end{array}$$

Moreover, note that if the equalities in (19) and (20) are attained, then *n* = 2, namely, *G*_1_ = *G*_2_ = *K*_2_. But $ℒEE(K_{2},K_{2})=e^{4}+1>\frac{2e^{2}(1+2e^{4})}{e^{4}+1}$, which means that the equality can not hold.

*Upper bound*. Again from the Neumann inequality, we arrive at

$$\begin{array}{ccc} ℒEE(G_{1},G_{2}) & = & \mathrm{tr}(e^{{ℒ}_{1}}e^{{ℒ}_{2}})\\ & \leq & \sum_{i=1}^{n}e^{\lambda_{i}\left({ℒ}_{1}\right)}e^{\lambda_{i}\left({ℒ}_{2}\right)}\\ & \leq & {\left(\sum_{i=1}^{n}e^{2\lambda_{i}\left({ℒ}_{1}\right)}\right)}^{\frac{1}{2}}\cdot {\left(\sum_{i=1}^{n}e^{2\lambda_{i}\left({ℒ}_{2}\right)}\right)}^{\frac{1}{2}}, \end{array}$$ (21)

where the last inequality follows from the Cauchy-Schwarz inequality.

Define the Randić index of a connected graph *G* as $R_{-1}(G)=\sum_{u,v\;\text{adjacent}}\deg_{G}^{-1}(u)\deg_{G}^{-1}(v)$. It is elementary that $\sum_{i=1}^{n}\lambda_{i}^{2}(ℒ(G))=n+2R_{-1}(G)$; see e.g. [50]. We have

$$\begin{array}{ccc} \sum_{i=1}^{n}e^{2\lambda_{i}\left({ℒ}_{1}\right)} & = & e^{2}\sum_{i=1}^{n}e^{2(\lambda_{i}\left({ℒ}_{1}\right)-1)}\\ & \leq & e^{2}\left(n+\sum_{i=1}^{n}\sum_{k=1}^{\infty }\frac{2^{k}{\left|\lambda_{i}\left({ℒ}_{1}\right)-1\right|}^{k}}{k!}\right)\\ & = & e^{2}\left(n+\sum_{k=1}^{\infty }\frac{2^{k}}{k!}\sum_{i=1}^{n}\left(\right|\lambda_{i}\left({ℒ}_{1}\right)-1{{|}^{2})}^{\frac{k}{2}}\right)\\ & \leq & e^{2}\left(n+\sum_{k=1}^{\infty }\frac{2^{k}}{k!}{\left(\sum_{i=1}^{n}{\left|\lambda_{i}\left({ℒ}_{1}\right)-1\right|}^{2}\right)}^{\frac{k}{2}}\right)\\ & = & e^{2}\left(n+\sum_{k=1}^{\infty }\frac{2^{k}}{k!}{\left(2R_{-1}\left(G_{1}\right)\right)}^{\frac{k}{2}}\right)\\ & = & e^{2}\left(n-1+e^{2\sqrt{2R_{-1}\left(G_{1}\right)}}\right). \end{array}$$ (22)

Since *G*_1_ is connected, we have [50]

$$\begin{array}{c} R_{-1}\left(G_{1}\right)\leq \frac{n}{2\delta (G_{1})}\leq \frac{n}{2}. \end{array}$$ (23)

Thus, (22) leads to the following estimation

$$\begin{array}{c} \sum_{i=1}^{n}e^{2\lambda_{i}\left({ℒ}_{1}\right)}\leq e^{2}\left(n-1+e^{2\sqrt{n}}\right). \end{array}$$

Combining this and an analogous estimation for ℒ_2_ yields the desired upper bound by using (21).

Finally, we note that the equalities in (23) hold if and only if *G*_1_ is a 1-regular graph, namely, $G_{1}=\underset{n/2\;\text{multiples}}{\underset{︸}{K_{2}\cup K_{2}\cdots \cup K_{2}}}$. But the first inequality in (22) is not tight for such choice of *G*_1_. Therefore, the equality in the upper bound can not be attained.

**Remark**. Recall that *δ*(*G*_*t*_) is the minimum degree of *G*_*t*_. The above proof actually gives a strong upper bound:

$$\begin{array}{c} ℒEE\left(G_{1},G_{2}\right)<e^{2}\cdot \sqrt{n-1+e^{2\sqrt{\frac{n}{\delta (G_{1})}}}}\cdot \sqrt{n-1+e^{2\sqrt{\frac{n}{\delta (G_{2})}}}}. \end{array}$$ (24)

**Proposition 6**. *The normalized Laplacian Estrada index of an evolving graph G*_1_, *G*_2_, ⋯, *G_N_ over a set of n* (*n* ≥ 2) *vertices with each snapshot graph being connected and N* = 2 *is bounded by*

$$\begin{array}{cc} ℒEE(G_{1},G_{2})< & \;e^{2}\cdot \sqrt{e^{2}+e^{-2}+n+1+e^{2\sqrt{\frac{n}{\delta (G_{1})}}}-2\sqrt{\frac{n}{\delta (G_{1})}}}\\ & \;\cdot \sqrt{e^{2}+e^{-2}+n+1+e^{2\sqrt{\frac{n}{\delta (G_{2})}}}-2\sqrt{\frac{n}{\delta (G_{2})}}}. \end{array}$$

**Proof**. As in the proof of Proposition 5, we have inequality (21).

Now that *G*_1_ is connected, we know that *λ*_*n*_(ℒ_1_) = 0, *λ*_1_(ℒ_1_) ≤ 2, and $\sum_{i=1}^{n}\lambda_{i}({ℒ}_{1})=n$ [19]. Therefore,

$$\begin{array}{ccc} \sum_{i=1}^{n}e^{2\lambda_{i}\left({ℒ}_{1}\right)} & = & e^{2}\sum_{i=1}^{n}e^{2(\lambda_{i}\left({ℒ}_{1}\right)-1)}\\ & \leq & e^{2}\left(e^{2}+e^{-2}+\sum_{i=2}^{n-1}e^{2(\lambda_{i}\left({ℒ}_{1}\right)-1)}\right)\\ & \leq & e^{2}\left(e^{2}+e^{-2}+n+2+\sum_{i=2}^{n-1}\sum_{k=2}^{\infty }\frac{2^{k}{\left|\lambda_{i}\left({ℒ}_{1}\right)-1\right|}^{k}}{k!}\right)\\ & \leq & e^{2}\left(e^{2}+e^{-2}+n+2+\sum_{k=2}^{\infty }\frac{2^{k}}{k!}{\left(\sum_{i=2}^{n-1}{\left|\lambda_{i}\left({ℒ}_{1}\right)-1\right|}^{2}\right)}^{\frac{k}{2}}\right)\\ & = & e^{2}\left(e^{2}+e^{-2}+n+2+\sum_{k=2}^{\infty }\frac{2^{k}}{k!}{\left(2R_{-1}\left(G_{1}\right)\right)}^{\frac{k}{2}}\right)\\ & = & e^{2}\left(e^{2}+e^{-2}+n+1+e^{2\sqrt{2R_{-1}\left(G_{1}\right)}}-2\sqrt{2R_{-1}\left(G_{1}\right)}\right). \end{array}$$ (25)

Define a function *f*(*x*) = *e* ^*x*^ − *x*, which is non-decreasing on [0, +∞). Thus, (23) and (25) indicate that

$$\begin{array}{c} \sum_{i=1}^{n}e^{2\lambda_{i}\left({ℒ}_{1}\right)}\leq e^{2}\left(e^{2}+e^{-2}+n+1+e^{2\sqrt{\frac{n}{\delta (G_{1})}}}-2\sqrt{\frac{n}{\delta (G_{1})}}\right). \end{array}$$

An analogous estimate for *G*_2_ also holds. Combining these with (21) yields the desired upper bound.

Finally, note that the second equality is attained in (25) if and only if *λ*_1_(ℒ_1_) = 0, which is equivalent to $G_{1}=\bar{K_{n}}$. However, this contradicts the assumption that *G*_1_ is connected. Therefore, the equality in the upper bound can not be attained. The proof is complete.

**Remark**. It is direct to check that if

$$\begin{array}{c} 2+e^{2}+e^{-2}<2\sqrt{\frac{n}{\delta (G_{1})}}\;\mathrm{and}\;2+e^{2}+e^{-2}<2\sqrt{\frac{n}{\delta (G_{2})}}, \end{array}$$

the above upper bound is better than that in (24).

Similarly as commented at the end of the above section, for the static case of *N* = 1, some bounds for the normalized Laplacian Estrada index are reported in the literature by involving more complicated graph-theoretic parameters, including normalized Laplacian energy [22], and the Randic index [35, 51], which are a bit cumbersome when large-scale network applications are taken into account.

### Numerical study

**Fig 1:**
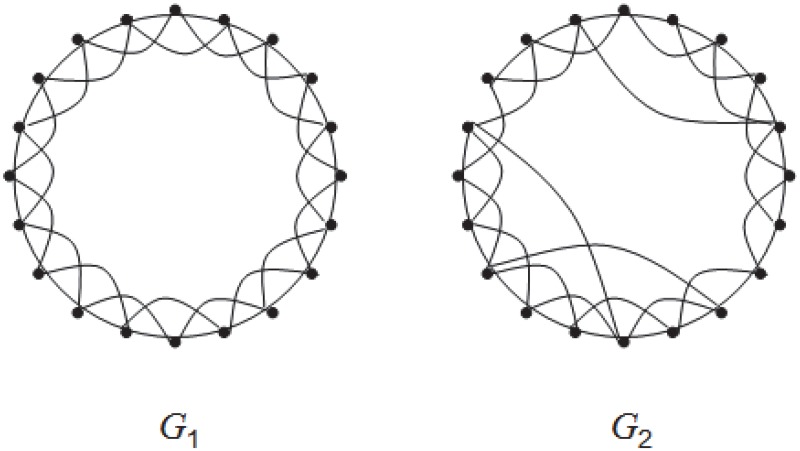
Illustration of an evolving small-world graph *G*_1_, *G*_2_. *G*_1_ is a ring lattice over a vertex set *V* of size *n*. It is a 4-regular graph, where each vertex is connected to its 4 nearest neighbors. *G*_2_ is obtained by rewiring each edge—i.e., choosing a vertex *v* ∈ *V* and an incident edge, reconnecting the edge to a vertex that is not a neighbor of *v*—with probability *p* = 0.01 uniformly at random. In the simulations below, we take *n* ∈ [100, 1000].

We consider a random evolving network *G*_1_, *G*_2_ (see Fig. 1), which is introduced in a seminal paper by Watts and Strogatz [52]. This network is often called WS small-world model, which enables the exploration of intermediate settings between purely local and purely global mixing. As demonstrated in [52], when the rewiring probability is taken around 0.01 (as we considered here), the model is highly clustered, like regular lattices, yet has small characteristic path lengths, like random graphs. This qualitative phenomenon is prevalent in a range of networks arising in nature and technology [53].

**Fig 2:**
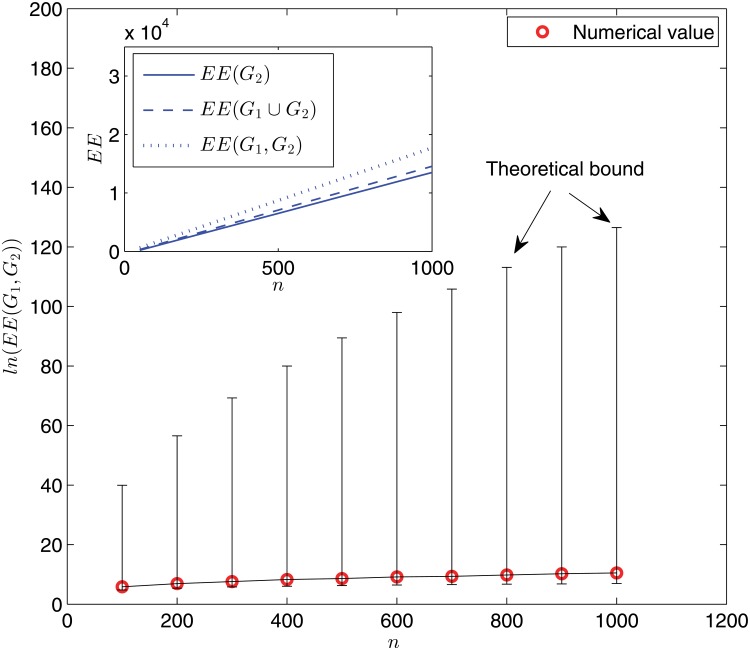
Logarithm of the dynamic Estrada index ln(*EE*(*G*_1_, *G*_2_)) as a function of network size *n*. Main panel: numerical results (red circles) and theoretical bounds (upper and lower bars) given by [18, Prop. 4]. Each data point is obtained for one network sample. Inset: simulation results for *EE*(*G*_1_, *G*_2_) (dotted line), *EE*(*G*_1_ ∪ *G*_2_) (dashed line), and *EE*(*G*_2_) (solid line) via an ensemble averaging of 100 independent random network samples.

Fig. 2 shows the variations of the (dynamic) Estrada indices with the network size *n*. The results gathered in Fig. 2 allow us to draw several interesting comments. First, as expected from the mathematical result [18, Prop. 4], the numerical values of *EE*(*G*_1_, *G*_2_) lie between our general upper and lower bounds (remarkably much closer to one than the other; see the main panel). Second, both the Estrada index and the dynamic Estrada index grow gradually as the network size increases. Third, the Estrada indices *EE*(*G*_2_) and *EE*(*G*_1_ ∪ *G*_2_) are close to each other. However, both of them are significantly smaller than the dynamic Estrada index *EE*(*G*_1_, *G*_2_), underscoring the relevance of dynamic Estrada index—neither the static snapshot graph nor the aggregated graph constitutes a reasonable approximation to the evolving graph itself.

**Fig 3:**
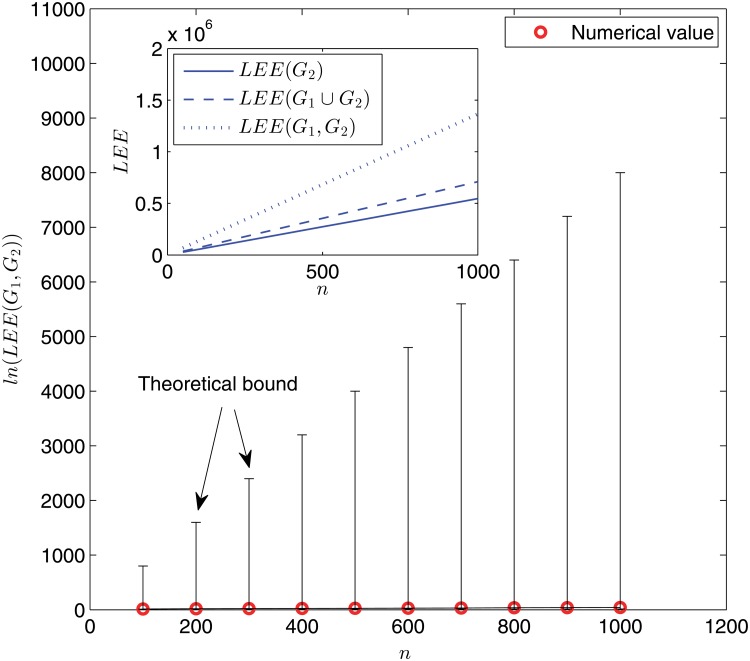
Logarithm of the dynamic Laplacian Estrada index ln(*LEE*(*G*_1_, *G*_2_)) as a function of network size *n*. Main panel: numerical results (red circles) and theoretical bounds (upper and lower bars) given by Proposition 2. Each data point is obtained for one network sample. Inset: simulation results for *LEE*(*G*_1_, *G*_2_) (dotted line), *LEE*(*G*_1_ ∪ *G*_2_) (dashed line), and *LEE*(*G*_2_) (solid line) via an ensemble averaging of 100 independent random network samples.

**Fig 4:**
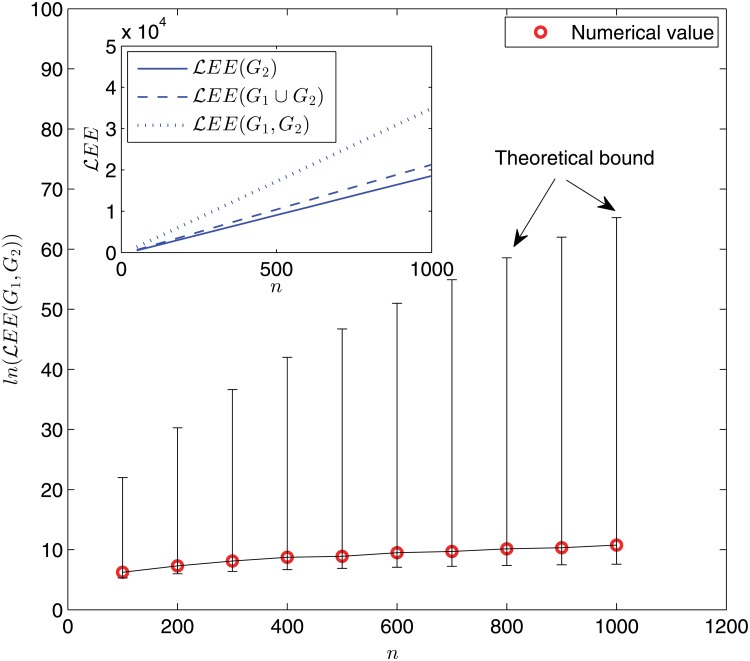
Logarithm of the dynamic normalized Laplacian Estrada index ln(ℒ*EE*(*G*_1_, *G*_2_)) as a function of network size *n*. Main panel: numerical results (red circles) and theoretical bounds (upper and lower bars) given by Proposition 5. Each data point is obtained for one network sample. Inset: simulation results for ℒ*EE*(*G*_1_, *G*_2_) (dotted line), ℒ*EE*(*G*_1_ ∪ *G*_2_) (dashed line), and ℒ*EE*(*G*_2_) (solid line) via an ensemble averaging of 100 independent random network samples.

In Fig. 3 and Fig. 4, we display the variations of the (dynamic) Laplacian Estrada indices and the (dynamic) normalized Laplacian Estrada indices, respectively, with the network size. Analogous observations can be drawn. For example, the behavior of *LEE*(*G*_1_, *G*_2_) (and ℒ*EE*(*G*_1_, *G*_2_)) differentiates from that of *LEE*(*G*_2_) (and ℒ*EE*(*G*_2_)) or *LEE*(*G*_1_ ∪ *G*_2_) (and ℒ*EE*(*G*_1_ ∪ *G*_2_)) dramatically. Moreover, when comparing Fig. 2 with Fig. 3 and Fig. 4, we see that the difference between dynamic and static cases turns out to be much more prominent in the Laplacian matrix and normalized Laplacian matrix settings than the adjacency matrix setting. For example, when the network size is taken as *n* = 1000, the difference ∣*EE*(*G*_1_, *G*_2_) − *EE*(*G*_1_ ∪ *G*_2_)∣ ≈ 4 × 10^3^; but ∣*LEE*(*G*_1_, *G*_2_) − *LEE*(*G*_1_ ∪ *G*_2_)∣ ≈ 7 × 10^5^ and ∣ℒ*EE*(*G*_1_, *G*_2_) − ℒ*EE*(*G*_1_ ∪ *G*_2_)∣ ≈ 1.4 × 10^4^.

Two remarks are in order. First, the theoretical upper and lower bounds for all the three dynamic Estrada indices shown in Figs. 2, 3, and 4 are fairly far apart, due to the fact that our bounds are general and valid for all graphs. This is similar to the situation of static graph case, see [12]. Thus, it would be interesting to identify the specific locations of concrete graphs (such as the WS small-world model studied here) in the spectrum. Second, extensive simulations have been performed for some different values of rewiring probability *p* and ring lattice degree *k*, all yielding quantitatively similar phenomena.

## Conclusion

A combined theoretical and computational analysis of the dynamic Estrada indices for evolving graphs has been performed. Following the dynamic Estrada index [18], (i) we investigated the dynamic Laplacian Estrada index and the dynamic normalized Laplacian Estrada index, whose mathematical properties such as the upper and lower bounds are established in general settings; (ii) the relations between bounds of these three dynamic Estrada indices are explored; (iii) the remarkable difference between static and dynamic indices are appreciated through numerical simulations for evolving random small-world networks.

The emergence of vast time-dependent networks in a range of fields demands the transition of analytic techniques from static graphs to evolving graphs. Many of these methods were reviewed in the surveys [2, 10]. We expect that the results developed in this paper can be used to evaluate various aspects of structure (in terms of graph spectra) and performance (such as robustness) of evolving networks. Some recent works relevant to the topic of Estrada index can be found in, e.g., [54–58].

## References

[1] Grindrod P, Higham DJ. Evolving graphs: dynamical models, inverse problems and propagation. Proc R Soc A Math Phys Eng Sci. 2010; 466: 753–770. 10.1098/rspa.2009.0456

[2] Holme P, Saramöki J. Temporal networks. Phys Rep. 2012; 519: 97–125. 10.1016/j.physrep.2012.03.001

[3] Grindrod P, Parsons MC, Higham DJ, Estrada E. Communicability across evolving networks. Phys Rev E. 2011; 83: 046120 10.1103/PhysRevE.83.046120 21599253

[4] Estrada E. Characterization of 3D molecular structure. Chem Phys Lett. 2000; 319: 713–718. 10.1016/S0009-2614(00)00158-5

[5] Estrada E. Characterization of the folding degree of proteins. Bioinformatics. 2002; 18: 697–704. 10.1093/bioinformatics/18.5.697 12050066

[6] Estrada E, Rodríguez-Velázquez JA, Randić M. Atomic branching in molecules. Int J Quantum Chem. 2006; 106: 823–832. 10.1002/qua.20850

[7] Estrada E, Rodríguez-Velázquez JA. Subgraph centrality in complex networks. Phys Rev E. 2005; 71: 056103 10.1103/PhysRevE.71.056103 16089598

[8] Estrada E, Rodríguez-Velázquez JA. Spectral measures of bipartivity in complex networks. Phys Rev E. 2005; 72: 046105 10.1103/PhysRevE.72.046105 16383466

[9] Shang Y. Perturbation results for the Estrada index in weighted networks. J Phys A Math Theor. 2011; 44: 075003 10.1088/1751-8113/44/7/075003

[10] Estrada E, Hatano N, Benzi M. The physics of communicability in complex networks. Phys Rep. 2012; 514: 89–119. 10.1016/j.physrep.2012.01.006 18517465

[11] de la Peñna JA, Gutman I, Rada J. Estimating the Estrada index. Linear Algebra Appl. 2007; 427: 70–76. 10.1016/j.laa.2007.06.020

[12] Gutman I, Deng H, Radenković S. The Estrada index: an updated survey In: Cvetković D, Gutman I, editors. Selected Topics on Applications of Graph Spectra. Beograd: Math Inst; 2011 pp. 155–174.

[13] Wu J, Barahona M, Tan Y, Deng H. Robustness of random graphs based on graph spectra. Chaos. 2012; 22: 043101 10.1063/1.4754875 23278036

[14] Wu J, Barahona M, Tan Y, Deng H. Natural connectivity of complex networks. Chin Phys Lett. 2010; 27: 078902 10.1088/0256-307X/27/7/078902

[15] Shang Y. Local natural connectivity in complex networks. Chin Phys Lett. 2011; 28: 068903 10.1088/0256-307X/28/6/068903

[16] Shang Y. Biased edge failure in scale-free networks based on natural connectivity. Indian J Phys. 2012; 86: 485–488. 10.1007/s12648-012-0084-4

[17] Shang Y. Random lifts of graphs: network robustness based on the Estrada index. Appl Math E-Notes. 2012; 12: 53–61.

[18] Shang Y. The Estrada index of evolving graphs. Appl Math Comput. 2015; 250: 415–423. 10.1016/j.amc.2014.10.129

[19] Chung FRK. Spectral Graph Theory. Providence: American Mathematical Society; 1997.

[20] Cvetković D, Doob M, Sachs H. Spectra of Graphs—Theory and Application. Heidelberg: Barth; 1995

[21] Fath-Tabar GH, Ashrafi AR, Gutman I. Note on Estrada and L-Estrada indices of graphs. Bull Acad Serbe Sci Arts (Cl Math Natur). 2009; 34: 1–16.

[22] Li J, Guo J, Shiu WC. The normalized Laplacian Estrada index of a graph. Filomat. 2014; 28: 365–371. 10.2298/FIL1402365L

[23] Estrada E. Communicability in temporal networks. Phys Rev E. 2013; 88: 042811 10.1103/PhysRevE.88.042811 24229229

[24] Grindrod P, Higham DJ. A matrix iteration for dynamic network summaries. SIAM Rev. 2013; 55: 118–128. 10.1137/110855715

[25] Grindrod P, Stoyanov ZV, Smith GM, Saddy JD. Primary evolving networks and the comparative analysis of robust and fragile structures. J Complex Networks. 2014; 2: 60–73. 10.1093/comnet/cnt015

[26] Grindrod P, Higham DJ. A dynamical systems view of network centrality. Proc R Soc A Math Phys Eng Sci. 2014; 470: 20130835 10.1098/rspa.2013.0835 PMC3973397 24808758

[27] Shang Y. Multi-agent coordination in directed moving neighborhood random networks. Chin Phys B. 2010; 19: 070201 10.1088/1674-1056/19/7/070201

[28] Li Q, Lqbal A, Perc M, Chen M, Abbott D. Coevolution of quantum and classical strategies on evolving random networks. PLoS One. 2013; 8: e68423 10.1371/journal.pone.0068423 23874622 PMC3709921

[29] Wu B, Zhou D, Fu F, Luo Q, Wang L, Traulsen A. Evolution of cooperation on stochastic dynamical networks. PLoS One. 2010; 5: e11187 10.1371/journal.pone.0011187 20614025 PMC2894855

[30] Shang Y. Estrada index of general weighted graphs. Bull Aust Math Soc. 2013; 88: 106–112. 10.1017/S0004972712000676

[31] Li J, Shiu WC, Chang A. On the Laplacian Estrada index of a graph. Appl Anal Discrete Math. 2009; 3: 147–156. 10.2298/AADM0901147L

[32] Huang F, Li X, Wang S. On maximum Laplacian Estrada indices of trees with some given parameters. MATCH Commun Math Comput Chem. 2015; 74: in press

[33] Azami S. On Laplacian and signless Laplacian Estrada indices of graphs. MATCH Commun Math Comput Chem. 2015; 74: in press

[34] Chen X, Hou Y. Some Results on Laplacian Estrada Index of Graphs. MATCH Commun Math Comput Chem. 2015; 73: 149–162.

[35] Hakimi-Nezhaad M, Hua H, shrafi AR, Qian S. The normalized Laplacian Estrada index of graphs. J Appl Math Informatics. 2014; 32: 227–245. 10.14317/jami.2014.227

[36] Shang Y. More on the normalized Laplacian Estrada index. Appl Anal Discrete Math. 2014; 8: 346–357. 10.2298/AADM140724011S

[37] Kuang J. Applied Inequalities. Jinan: Shandong Science and Technology Press; 2004.

[38] Bellman R. Introduction to Matrix Analysis. New York: McGraw-Hill; 1970.

[39] Gutman I, Das KC. The first Zagreb index 30 years after. MATCH Commun Math Comput Chem. 2004; 50: 83–92.

[40] Shang Y. Lower bounds for the Estrada index using mixing time and Laplacian spectrum. Rocky Mountain J Math. 2013; 43: 2009–2016. 10.1216/RMJ-2013-43-6-2009

[41] Zhou B, Gutman I. More on the Laplacian Estrada index. Appl Anal Discrete Math. 2009; 3: 371–378. 10.2298/AADM0902371Z

[42] Li X, Shi Y, Gutman I. Graph Energy. New York: Springer; 2012.

[43] Huo B, Li X, Shi Y. Complete solution to a conjecture on the maximal energy of unicyclic graphs. European J Combin. 2011; 32: 662–673. 10.1016/j.ejc.2011.02.011

[44] Gutman I, Zhou B. Laplacian energy of a graph. Linear Algebra Appl. 2006; 414: 29–37. 10.1016/j.laa.2005.09.008

[45] Huo B, Li X, Shi Y. Complete solution to a problem on the maximal energy of unicyclic bipartite graphs. Linear Algebra Appl. 2011; 434: 1370–1377. 10.1016/j.laa.2010.11.025

[46] Bollobás B, Erdős P. Graphs of extremal weights. Ars Combin. 1998; 50: 225–233

[47] Hu Y, Li X, Shi Y, Xu T, Gutman I. On molecular graphs with smallest and greatest zeroth-order general Randić index. MATCH Commun Math Comput Chem. 2005; 54: 425–434.

[48] Hu Y, Li X, Shi Y, Xu T. Connected (*n*, *m*)-graphs with minimum and maximum zeroth-order general Randić index. Discrete Appl Math. 2007; 155: 1044–1054. 10.1016/j.dam.2006.11.008

[49] Wang CL. On development of inverses of Cauchy and Hölder inequalities. SIAM Rev. 1979; 21: 550–557. 10.1137/1021096

[50] Cavers M. The normalized Laplacian matrix and general Randić index of graphs. Ph.D. Thesis, University of Regina. 2010.

[51] Li X, Shi Y. A survey on the Randić index. MATCH Commun Math Comput Chem. 2008; 59: 127–156.

[52] Watts DJ, Strogatz SH. Collective dynamics of ‘small-world’ networks. Nature. 1998; 393: 440–442. 10.1038/30918 9623998

[53] Albert R, Barabási A-L. Statistical mechanics of complex networks. Rev Mod Phys. 2002; 74: 47–97. 10.1103/RevModPhys.74.47

[54] Gao N, Qiao L, Ning B, Zhang S. Coulson-type integral formulas for the Estrada index of graphs and the skew Estrada index of oriented graphs. MATCH Commun Math Comput Chem. 2015; 73: 133–148.

[55] Chen L, Shi Y. Maximal matching energy of tricyclic graphs. MATCH Commun Math Comput Chem. 2015; 73: 105–119.

[56] Chen X, Qian J. On resolvent Estrada index. MATCH Commun Math Comput Chem. 2015; 73: 163–174.

[57] Gutman I, Furtula B, Chen X, Qian J. Graphs with smallest resolvent Estrada indices. MATCH Commun Math Comput Chem. 2015; 73: 267–270.

[58] Shang Y. Distance Estrada index of random graphs. Linear Multilinear Algebra. 2015; 63: 466–471. 10.1080/03081087.2013.872640

